# Supplementary material for: Age-dependent dormant resident progenitors are stimulated by injury to regenerate Purkinje neurons
Source: eLife. 2018 Aug 9;7:e39879. doi: 10.7554/eLife.39879 (PMC6115187; doi:10.7554/eLife.39879)
Supplement: Figure 1—source data 1. [file elife-39879-fig1-data1.docx]

**Figure 1_source data 1. Summary of the antibodies used in the study.**

| **Target** | **Catalog Number** | **Company** | **Dilution** |
| --- | --- | --- | --- |
| **Goat α-FoxP2** | EB05226 | Everest | 1/1000 |
| **Rabbit α-Calbindin1** | CB38 | Swant | 1/1000 |
| **Mouse α-Calbindin1** | 300 | Swant | 1/1000 |
| **Goat α-Sox2** | AF2018 | R&D System | 1/500 |
| **Rabbit α-Pax2** | 71600 | Invitrogen | 1/500 |
| **Rabbit α-Pax6** | AB2237 | Millipore | 1/500 |
| **Rabbit α-Ki67** | RM-9106-S0 | Thermo Scientific | 1/500 |
| **Rat α-BrdU** | OBT0030CX | Accurate | 1/500 |
| **Sheep α-BrdU** | ab1893 | Abcam | 1/500 |
| **Mouse α-p27** | 610241 | BD Pharmingen | 1/500 |
| **Rat α-GFP (CFP)** | 04404-84 | Nacalai Tesque | 1/1000 |
| **γ-H2AX(Ser139)** | 9718 | Cell Signaling | 1/250 |
| **Mouse γ-tubulin (GTU-88)** | 6557 | Sigma | 1/1000 |
| **Goat α-hHB-EGF(DTR)** | AF231 | R&D System | 1/500 |
