# Supplementary material for: Age-dependent dormant resident progenitors are stimulated by injury to regenerate Purkinje neurons
Source: eLife. 2018 Aug 9;7:e39879. doi: 10.7554/eLife.39879 (PMC6115187; doi:10.7554/eLife.39879)
Supplement: Figure 1—source data 2. [file elife-39879-fig1-data2.docx]

**Figure 1_source data 2. Summary of the statistics performed**

| **Figure** | **Test performed** | **P-Value** | **Multiple comparisons** | |  |
| --- | --- | --- | --- | --- | --- |
| Figure 1P | Two-way ANOVA | PCL cells: F_(5,54)_=4.034, P=0.0035  total number of PCs: F_(5,27)_=4.732, P=0.003 | P2:  No DT (PCL) vs. DT@P1 (PCL) | 0.0258 | |
|  |  |  | P2:  No DT (PCL) vs. DT@P1 (Ectopic) | <0.0001 |  |
|  |  |  | P2:  DT@P1 (PCL) vs. DT@P1 (Ectopic) | 0.0212 |  |
|  |  |  | P3:  No DT (PCL) vs. DT@P1 (PCL) | 0.0442 |  |
|  |  |  | P3:  No DT (PCL) vs. DT@P1 (Ectopic) | <0.0001 |  |
|  |  |  | P3:  DT@P1 (PCL) vs. DT@P1 (Ectopic) | 0.0982 |  |
|  |  |  | P5:  No DT (PCL) vs. DT@P1 (PCL) | 0.4370 |  |
|  |  |  | P5:  No DT (PCL) vs. DT@P1 (Ectopic) | <0.0001 |  |
|  |  |  | P5:  DT@P1 (PCL) vs. DT@P1 (Ectopic) | <0.0001 |  |
|  |  |  | P8:  No DT (PCL) vs. DT@P1 (PCL) | 0.7943 |  |
|  |  |  | P8:  No DT (PCL) vs. DT@P1 (Ectopic) | <0.0001 |  |
|  |  |  | P8:  DT@P1 (PCL) vs. DT@P1 (Ectopic) | <0.0001 |  |
|  |  |  | P12:  No DT (PCL) vs. DT@P1 (PCL) | 0.7872 |  |
|  |  |  | P12:  No DT (PCL) vs. DT@P1 (Ectopic) | <0.0001 |  |
|  |  |  | P12:  DT@P1 (PCL) vs. DT@P1 (Ectopic) | <0.0001 |  |
|  |  |  | P30:  No DT (PCL) vs. DT@P1 (PCL) | 0.6417 |  |
|  |  |  | P30:  No DT (PCL) vs. DT@P1 (Ectopic) | <0.0001 |  |
|  |  |  | P30:  DT@P1 (PCL) vs. DT@P1 (Ectopic) | <0.0001 |  |
| Figure 1Q | Two-way ANOVA | PCL cells: F_(5,8)_=6.957, P=0.0001 | P2:  No DT (PCL) vs. DT@P1 (PCL) | 0.0003 |  |
|  |  |  | P2:  No DT (PCL) vs. DT@P1 (Ectopic) | 0.3741 |  |
|  |  |  | P2:  DT@P1 (PCL) vs. DT@P1 (Ectopic) | 0.0144 |  |
|  |  |  | P3:  No DT (PCL) vs. DT@P1 (PCL) | <0.0001 |  |
|  |  |  | P3:  No DT (PCL) vs. DT@P1 (Ectopic) | 0.9674 |  |
|  |  |  | P3:  DT@P1 (PCL) vs. DT@P1 (Ectopic) | <0.0001 |  |
|  |  |  | P5:  No DT (PCL) vs. DT@P1 (PCL) | 0.0297 |  |
|  |  |  | P5:  No DT (PCL) vs. DT@P1 (Ectopic) | 0.8486 |  |
|  |  |  | P5:  DT@P1 (PCL) vs. DT@P1 (Ectopic) | 0.1587 |  |
|  |  |  | P8:  No DT (PCL) vs. DT@P1 (PCL) | 0.0019 |  |
|  |  |  | P8:  No DT (PCL) vs. DT@P1 (Ectopic) | <0.0001 |  |
|  |  |  | P8:  DT@P1 (PCL) vs. DT@P1 (Ectopic) | 0.6641 |  |
|  |  |  | P12:  No DT (PCL) vs. DT@P1 (PCL) | 0.0193 |  |
|  |  |  | P12:  No DT (PCL) vs. DT@P1 (Ectopic) | 0.0006 |  |
|  |  |  | P12:  DT@P1 (PCL) vs. DT@P1 (Ectopic) | 0.2535 |  |
|  |  |  | P30:  No DT (PCL) vs. DT@P1 (PCL) | 0.0273 |  |
|  |  |  | P30:  No DT (PCL) vs. DT@P1 (Ectopic) | <0.0001 |  |
|  |  |  | P30:  DT@P1 (PCL) vs. DT@P1 (Ectopic) | 0.0111 |  |
| Figure 1T |  | \|  \| \| --- \|   F_(1, 32)_=3.043, P=0.89 | P1.5 (No DT vs. DT@P1) | 0.9997 |  |
|  |  |  | P2 (No DT vs. DT@P1) | >0.9999 |  |
|  |  |  | P3 (No DT vs. DT@P1) | >0.9999 |  |
|  |  |  | P5 (No DT vs. DT@P1) | 0.9984 |  |
|  |  |  | P8 (No DT vs. DT@P1) | 0.9976 |  |
|  |  |  | P12 (No DT vs. DT@P1) | 0.4230 |  |
|  |  |  | P30 (No DT vs. DT@P1) | 0.9201 |  |
| Figure 2B | Two-way ANOVA | F_(3,16)_=6.163, P=0.006 | 4-8h post injection vs. 10-14h post injection | 0.0037 |  |
|  |  |  | 4-8h post injection vs. 16-20h post injection | 0.0051 |  |
|  |  |  | 4-8h post injection vs. 22-24h post injection | >0.9999 |  |
|  |  |  | 10-14h post injection vs. 16-20h post injection | \| >0.9999 \| \| --- \| |  |
|  |  |  | 10-14h post injection vs. 22-24h post injection | \| 0.0022 \| \| --- \| |  |
|  |  |  | 16-20h post injection vs. 22-24h post injection | \| 0.0030 \| \| --- \| |  |
| Figure 3C | CALB1+ cells: One-way ANOVA | F_(2.6)_=6.883, P=0.028 | P1 vs. P5 | 0.4160 |  |
|  |  |  | P1 vs. P30 | 0.0244 |  |
|  |  |  | P5 vs. P30 | 0.1304 |  |
| Figure 3C | iPCs: Student’s two tailed t-test | t(4)=8.904, P=0.0009 | N/A | N/A |  |
| Figure 3C | All cells: One-way ANOVA | F_(2.6)_=1.813, P=0.24 | N/A | N/A |  |
| Figure 3D | Two-tailed  t-test | P1.5: t(4)=5.523, P=0.005  P5: t(4)=2.955, P=0.04 | N/A | |  |
| Figure 4A | One-way ANOVA | F_(2,16)_=9.464, P=0.002 | No DT vs. DT@P1 | 0.6462 |  |
|  |  |  | No DT vs. DT@P5 | 0.0019 |  |
|  |  |  | DT@P1 vs. DT@P5 | 0.0155 |  |
| Figure 4B | Two-tailed t-test | t(4)=3.301, P=0.04 | N/A | |  |
| Figure 4C | Two-way ANOVA | F_(1,22)_=7.045, P=0.01 | P8 | 0.9946 |  |
|  |  |  | P12 | 0.0121 |  |
|  |  |  | P16 | 0.2788 |  |
|  |  |  | P30 | 0.8979 |  |
| Figure 4D | One-way ANOVA | F_(2,11)_=20.56, P=0.0002 | No DT vs. DT@P1 | 0.7328 |  |
|  |  |  | No DT vs. DT@P5 | 0.0003 |  |
|  |  |  | DT@P1 vs. DT@P5 | 0.0013 |  |
| Figure 4E | One-way ANOVA | F_(2,11)_=14.54, P=0.0008 | No DT vs. DT@P1 | 0.8645 |  |
|  |  |  | No DT vs. DT@P5 | 0.0011 |  |
|  |  |  | DT@P1 vs. DT@P5 | 0.0041 |  |
| Figure 4F | Two-way ANOVA | F_(2,34)_=8.37, P=0.001 | day1 trial 1:  No DT vs. DT@P1  No DT vs. DT@P5  DT@P1 vs. DT@P5 | \| 0.9895 \| \| --- \| \| 0.8013 \| \| 0.7792 \| |  |
|  |  |  | day1 trial 2:  No DT vs. DT@P1  No DT vs. DT@P5  DT@P1 vs. DT@P5 | \| 0.6132 \| \| --- \| \| 0.0773 \| \| 0.5734 \| |  |
|  |  |  | day1 trial 3:  No DT vs. DT@P1  No DT vs. DT@P5  DT@P1 vs. DT@P5 | \| 0.4090 \| \| --- \| \| 0.0005 \| \| 0.0941 \| |  |
|  |  |  | Day2 trial 1:  No DT vs. DT@P1  No DT vs. DT@P5  DT@P1 vs. DT@P5 | \| 0.9765 \| \| --- \| \| 0.0315 \| \| 0.1161 \| |  |
|  |  |  | Day2 trial 2:  No DT vs. DT@P1  No DT vs. DT@P5  DT@P1 vs. DT@P5 | \| 0.5224 \| \| --- \| \| 0.0093 \| \| 0.2694 \| |  |
|  |  |  | Day2 trial 3:  No DT vs. DT@P1  No DT vs. DT@P5  DT@P1 vs. DT@P5 | \| 0.5429 \| \| --- \| \| 0.1141 \| \| 0.7316 \| |  |
|  |  |  | Day3 trial 1:  No DT vs. DT@P1  No DT vs. DT@P5  DT@P1 vs. DT@P5 | \| 0.9993 \| \| --- \| \| 0.0029 \| \| 0.0141 \| |  |
|  |  |  | Day3 trial 2:  No DT vs. DT@P1  No DT vs. DT@P5  DT@P1 vs. DT@P5 | \| 0.8835 \| \| --- \| \| 0.0192 \| \| 0.0163 \| |  |
|  |  |  | Day3 trial 3:  No DT vs. DT@P1  No DT vs. DT@P5  DT@P1 vs. DT@P5 | \| 0.7927 \| \| --- \| \| 0.0002 \| \| 0.0117 \| |  |
| Figure 4G | One-way ANOVA | F_(2,34)_=8.37, P=0.001 | No DT vs. DT@P1 | 0.8290 |  |
|  |  |  | No DT vs. DT@P5 | 0.0009 |  |
|  |  |  | DT@P1 vs. DT@P5 | 0.0280 |  |
| Figure 4H | One-way ANOVA | F_(2,34)_=0.4181, P=0.66 | No DT vs. DT@P1 | 0.8869 |  |
|  |  |  | No DT vs. DT@P5 | 0.8430 |  |
|  |  |  | DT@P1 vs. DT@P5 | 0.6382 |  |
| Figure 4J | Two-way ANOVA | No DT vs. DT@P5:  F_(2,133)_=73.45, P=0.0001 | Stride:  No DT vs. DT@P1  No DT vs. DT@P5  DT@P1 vs. DT@P5 | \| 0.0706 \| \| --- \| \| <0.0001 \| \| <0.0001 \| |  |
|  |  |  | Sway:  No DT vs. DT@P1  No DT vs. DT@P5  DT@P1 vs. DT@P5 | \| 0.7319 \| \| --- \| \| 0.7312 \| \| 0.3750 \| |  |
| Figure 1_supplement 1F | One-way ANOVA | F_(2,9)_=04341, P=0.66 | P1 vs. P5 | 0.7156 |  |
|  |  |  | P1 vs. P30 | 0.9961 |  |
|  |  |  | P5 vs. P30 | 0.7304 |  |
| Figure 1_supplement 2I | Two-way ANOVA | F_(1,40)_=4.847, P=0.033 | P1.5 | 0.6225 |  |
|  |  |  | P2 | >0.9999 |  |
|  |  |  | P3 | 0.8429 |  |
|  |  |  | P5 | 0.8241 |  |
|  |  |  | P8 | 0.9998 |  |
|  |  |  | P12 | 0.7582 |  |
|  |  |  | P30 | >0.9999 |  |
| Figure 1_supplement 3I | Two-way ANOVA | F_(1,24)_=0.03658, P=0.8499 | N/A | N/A |  |
| Figure 3_supplement 1 | One-way ANOVA | F_(3,9)_=9.074, P=0.004 | P1 vs. P2 | 0.6249 |  |
|  |  |  | P1 vs. P3 | 0.0259 |  |
|  |  |  | P1 vs. P5 | 0.0050 |  |
|  |  |  | P2 vs. P3 | 0.1830 |  |
|  |  |  | P2 vs. P5 | 0.0293 |  |
|  |  |  | P3 vs. P5 | 0.4893 |  |
| Figure 3_supplement 6B | Student’s t-test | t_(45)_=3.593, P=0.0008 | N/A | N/A |  |
| Figure 3_supplement 6D | Student’s t-test | t_(73)_=4.11, P=0.0001 | N/A | N/A |  |
| Figure 4_supplement 1R | Two-way ANOVA | F_(1,24)_=77.85, P=0.0001 | P8 (No DT vs. DT@P5) | 0.0532 |  |
|  |  |  | P12 (No DT vs. DT@P5) | <0.0001 |  |
|  |  |  | P16 (No DT vs. DT@P5) | 0.0002 |  |
|  |  |  | P30 (No DT vs. DT@P5) | 0.0001 |  |
| Figure 4_supplement 1S | Two-way ANOVA | F_(1,21)_=40.1, P=0.0001 | P8 (No DT vs. DT@P5) | 0.2620 |  |
|  |  |  | P12 (No DT vs. DT@P5) | 0.0654 |  |
|  |  |  | P16 (No DT vs. DT@P5) | 0.0002 |  |
|  |  |  | P30 (No DT vs. DT@P5) | 0.0345 |  |
| Figure 4_Supplement 3I | One-way ANOVA | F_(1,22)_=.7.799, P=0.01 | P8 (No DT vs. DT@P5) | 0.9665 |  |
|  |  |  | P12 (No DT vs. DT@P5) | 0.0083 |  |
|  |  |  | P16 (No DT vs. DT@P5) | 0.3611 |  |
|  |  |  | P30 (No DT vs. DT@P5) | 0.9236 |  |
| Figure 4_supplement 3J | One-way ANOVA | F(2,12)=9.687, P=0.003 | No DT vs. DT@P1 | 0.7130 |  |
|  |  |  | No DT vs. DT@P5 | 0.0030 |  |
|  |  |  | DT@P1 vs. DT@P5 | 0.0242 |  |
| Figure 4_supplement 4E | Two-tailed  t-test | P8: t(6)=2.452, P=0.049  P12: t(6)=0.7087, P=0.5 | N/A | N/A |  |
| Figure 4_supplement 4F | One-way ANOVA | F_(2,12)_=15.73, P=0.0004 | No DT vs. DT@P1 | 0.3819 |  |
|  |  |  | No DT vs. DT@P5 | 0.0026 |  |
|  |  |  | DT@P1 vs. DT@P5 | 0.0006 |  |
